# A DPP-4 Inhibitor Suppresses Fibrosis and Inflammation on Experimental Autoimmune Myocarditis in Mice

**DOI:** 10.1371/journal.pone.0119360

**Published:** 2015-03-13

**Authors:** Hiroyuki Hirakawa, Hirofumi Zempo, Masahito Ogawa, Ryo Watanabe, Jun-ichi Suzuki, Hiroshi Akazawa, Issei Komuro, Mitsuaki Isobe

**Affiliations:** 1 Department of Cardiovascular Medicine, Tokyo Medical and Dental University, Tokyo, Japan; 2 Department of Advanced Clinical Science and Therapeutics, The University of Tokyo, Tokyo, Japan; 3 Department of Cardiovascular Medicine, The University of Tokyo, Tokyo, Japan; Osaka University Graduate School of Medicine, JAPAN

## Abstract

Myocarditis is a critical inflammatory disorder which causes life-threatening conditions. No specific or effective treatment has been established. DPP-4 inhibitors have salutary effects not only on type 2 diabetes but also on certain cardiovascular diseases. However, the role of a DPP-4 inhibitor on myocarditis has not been investigated. To clarify the effects of a DPP-4 inhibitor on myocarditis, we used an experimental autoimmune myocarditis (EAM) model in Balb/c mice. EAM mice were assigned to the following groups: EAM mice group treated with a DPP-4 inhibitor (linagliptin) (n = 19) and those untreated (n = 22). Pathological analysis revealed that the myocardial fibrosis area ratio in the treated group was significantly lower than in the untreated group. RT-PCR analysis demonstrated that the levels of mRNA expression of IL-2, TNF-α, IL-1β and IL-6 were significantly lower in the treated group than in the untreated group. Lymphocyte proliferation assay showed that treatment with the DPP-4 inhibitor had no effect on antigen-induced spleen cell proliferation. Administration of the DPP-4 inhibitor remarkably suppressed cardiac fibrosis and reduced inflammatory cytokine gene expression in EAM mice. Thus, the agents present in DPP-4 inhibitors may be useful to treat and/or prevent clinical myocarditis.

## Introduction

Myocarditis is a critical inflammatory disorder which causes severe left ventricular (LV) dysfunction, life-threatening arrhythmia and cardiac sudden death in the acute phase [1]. Severe inflammation response in the myocardium also triggers cardiac remodeling. This is associated with an increased risk of the development of dilated cardiomyopathy (DCM) by inducing cardiac fibrosis, accumulation of extracellular matrix, cardiomyocyte apoptosis, and decreased contractility [2–4]. Previous investigations showed that there are several potential targets in myocarditis [4], however, no specific and effective treatment of myocarditis has been established. Thus, developing new therapeutic strategies which aim to alleviate myocarditis are required.

Dipeptidyl peptidase-4 (DPP-4), a 766-amino-acid protein, is an amino peptidase which cleaves two amino acids from the N-terminus of target proteins or peptides, thereby reducing the activity of its substrates [5, 6]. A major enzymatic function of DPP-4 is reducing the activity of incretin peptides, including glucagon-like peptide-1 (GLP-1) and glucose-dependent insulinotropic polypeptide (GIP), which contributes to the attenuation of insulin secretion [6, 7]. DPP-4 inhibitors have been broadly used as a therapeutic agent for type 2 diabetes.

Accumulating lines of evidence suggest the beneficial effects of DPP-4 inhibitors include not only type 2 diabetes but also various types of cardiovascular diseases. Administration of alogliptin, a DPP-4 inhibitor, decreased reactive oxygen species (ROS) and effectively attenuated aortic aneurysmal formation [8]. Administration of linagliptin, another DPP-4 inhibitor, significantly reduced the infracted area after cerebral infarction [9]. The DPP-4 inhibitors effect on cardiovascular disease led us to hypothesize that it also has beneficial effects on myocarditis. Thus, the aim of this study was to clarify the role of DPP-4 inhibitors in murine experimental autoimmune myocarditis (EAM).

## Methods

### Animals and Immunization

Male Balb/c mice (6-weeks-old; body weight 20 to 25g) were purchased from CLEA JAPAN. They were fed a standard diet. All animal procedures were carried out in accordance with the recommendations in the Guide for the Care and Use of Laboratory Animals of the National Institutes of Health. This protocol was approved by the Committee on the Ethics of Animal Experiments of Tokyo Medical and Dental University (Permit Number: 0140140A). MyHC-α^614–629^ (Japan Bio Service Co., Saitama, Japan), a purified synthetic peptide, was emulsified with an equal volume of complete Freund’s adjuvant supplemented with *Mycobacterium tuberculosis* H37RA (Difco, sparks, MD, US) [10, 11]. We injected 0.2mL of emulsion (150 μg of cardiac myosin per mouse) using a 27-gauge needle under the dorsal skin of each mouse on day 0 and 7 to induce EAM [10]. Mice were anesthetized with 2, 2, 2- trichloro-1, 1-ethanediol and all efforts were made to minimize suffering.

### A DPP-4 Inhibitor Administration

Linagliptin, a DPP-4 inhibitor, was provided by Boehringer Ingelheim (Ingelheim, Germany), with a material transfer agreement. The immunized mice were randomly assigned to two groups. Based on the previous report [12], the treated group (n = 19) was orally administrated linagliptin (1.0 mg/kg) from day 0 to day 21 and the untreated group (n = 22) was orally administrated vehicle from day 0 to day 21. Unimmunized mice without linagliptin administration were used as a control group.

### Echocardiogram

Transthoracic echocardiography was performed on the animals anesthetized by intra-peritoneal administration of 3.6% chloral hydrate (2,2,2-trichloro-1,1,ethanediol: Wako Pure Chemical Industries, Osaka, Japan) in saline on day 21. An echocardiography machine with a 14MHz transducer (Toshiba, Tokyo, Japan) was used for M-mode LV echocardiographic recording. A two dimensional targeted M-mode echocardiogram was obtained along the short-axis view of the LV at the papillary muscles. LV fractional shortening was calculated from M-mode echocardiograms over three consecutive cardiac cycles according to the American Society for Echocardiography leading edge method [2, 13]. A B-mode echocardiogram was used to observe the LV function. To analyze cardiac function, two investigators measured the contraction independently, and the values were averaged.

### Histopathology

Hearts were harvested immediately after all animals were sacrificed by the cutting of the abdominal aorta under deep anesthesia on day 21. After measuring the weight (mg), several sections per heart were obtained for histological examination. Apex, midventricular, and basal level slices were stained with hematoxylin and eosin (HE) and Mallory staining. The area of the myocardium affected by cell infiltration was determined as infiltrated areas; fibrosis and necrotic changes were calculated as fibrosis areas. We calculated the area ratio (cell infiltration or fibrosis areas/entire area) [14]. To avoid bias, two independent investigators, who were blinded to the slide identification, used Image-J (National Institutes of Health, USA), a computer analysis system, to measure the cell infiltrating and fibrosis areas. The values were averaged.

### Immunohistochemistry

Immunohistochemistry was performed to examine CD4 (#1540–10, SouthernBiotech), F4/80 (#123102, BioLegend) expression in the hearts on day 21. Frozen sections were fixed in acetone at 4°C. The sections were incubated with unlabelled primary antibodies over night at 4°C and washed in PBS. We then applied secondary antibodies (Histofine; Nichirei Co., Tokyo, Japan) at 5μg/mL for 60 min at room temperature. The sections were washed in PBS and incubated with an aminoethylcarbazole (AEC) complex (Nichirei). Sections were then counterstained with hematoxylin solution (Sigma-Aldrich Japan, Tokyo, Japan). CD4 and F4/80 positive cells were counted and the number was divided by the entire area.

### Real-time RT-PCR

Total RNA was isolated from the hearts and cDNA was prepared with the RT-PCR Kit (Lifetechnologies Japan, Tokyo, Japan). Using Real-time PCR in a StepOne real-time PCR system (Lifetechnologies Japan, Tokyo, Japan), the mRNA expression of interleukin (IL)-2 (Mm00434256_m1), tumor necrosis factor (TNF)-α (Mm00443258_m1), IL-1β (Mm00434228_m1), IL-6 (Mm00446190_m1), collagen (Col)-1a1 (Mm00801666_g1), and fibronectin (Fn)-1 (Mm01256744_m1) were determined. A real-time PCR protocol was performed using the following cycling parameters: 95°C for 20 sec followed by 50 cycles: 95°C for 1 sec, annealing at 60°C for 20 sec. cDNA was run in duplicates and quantitative data was calculated using the comparative Ct (ΔΔCt) method. mRNA levels were quantified and normalized against levels of 18s (4319413E).

### Lymphocyte Proliferation Assay

Spleen cells were isolated aseptically from mice with EAM on day 21. After removing red blood cells, splenic lymphocytes (2.5x10^5^/well) were incubated in RPMI 1640 medium supplemented with 10% fetal bovine serum (FBS) and 10–4% 2-mercaptoethanol (Katayama Chemical Japan, Osaka, Japan) at 37°C with 5% CO_2_ for 1 hour. Then, 100μl MyHC-α614–629 (50μg/ml) was added to the cultures. We applied linagliptin to each well at various concentrations. Cultures were incubated at 37°C with 5% CO_2_ for 70 hours. Lymphocyte proliferation was estimated with a cell counting kit-8 (Dojindo, Kumamoto, Japan) [15]. Cell proliferation was expressed as optical density 45 minutes after adding the cell counting kit-8.

### Statistical Analysis

Values were given as mean ± standard error of mean. Group comparisons were performed by unpaired T test (for echocardiogram and immunohistochemistry), and one-way ANOVA followed by either Fisher’s PLSD (for pathology and PCR) or the Bonferroni (for lymphocyte proliferation assay) comparison test. Differences were considered statistically significant at a value of P<0.05.

## Results

### Heart and Lung Weights

Heart weight to body weight (mg/g) was not significantly different between the treated group (4.73 ± 0.12) and the untreated group (4.95 ± 0.13). Lung weight to body weight (mg/g), an index of lung congestion, was not significantly different between the treated group (4.85 ± 0.20) and the untreated group (5.09 ± 0.23).

### Cardiac Function

Echocardiographic measurements indicated that fractional shortening (FS), ejection fraction (EF), interventricular septal thickness at end-diastole (IVSTd), LV internal dimension diastolic (LVIDd), LV posterior wall diastolic (LVPWd), and LV internal dimension systolic (LVIDs) were not significantly different between the treated group and the untreated group. (Table 1)

**Table 1:** Echocardiography on day 21.

	Vehicle	Linagliptin	P value
	(n = 8)	(n = 6)	
FS (%)	48.7 ± 1.0	48.3 ± 1.9	0.86
EF (%)	85.4 ± 0.8	85.0 ± 1.8	0.81
IVSTd (mm)	0.94 ± 0.02	0.94 ± 0.05	0.92
LVIDd (mm)	3.34 ± 0.06	3.29 ± 0.15	0.75
LVPWd (mm)	0.94 ± 0.04	0.93 ± 0.04	0.94
LVIDs (mm)	1.70 ± 0.06	1.69 ± 0.04	0.91

FS, fractional shortening; EF, ejection fraction; IVSTd, interventricular septal thickness at end-diastole; LVIDd, left ventricular internal dimension diastolic; LVPWd, left ventricular posterior wall diastolic; LVIDs, left ventricular internal dimension systolic.

### Histopathology

**Fig 1:**
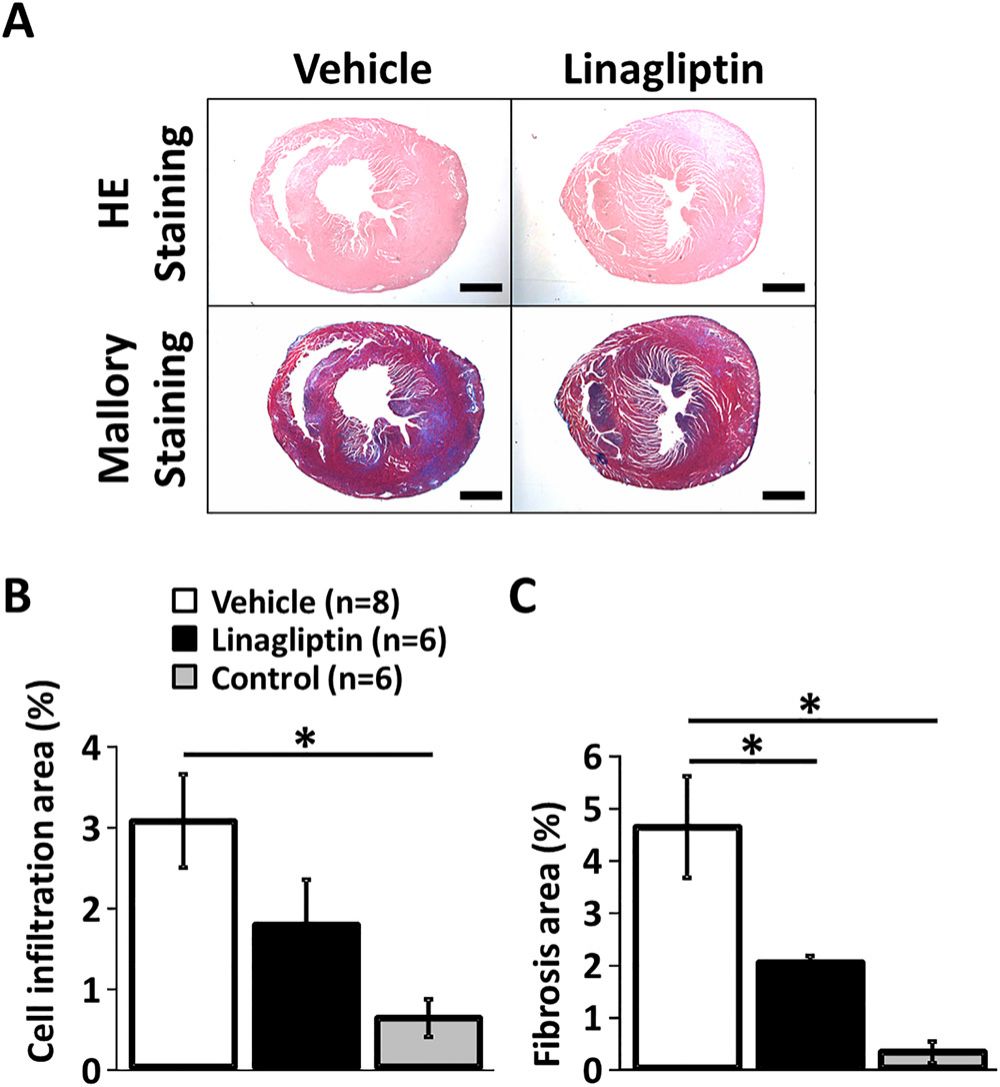
Pathological analysis revealed that a DPP-4 inhibitor prevented cardiac fibrosis in EAM. A, Representative pathological images stained with HE staining and Mallory staining in low (× 20) power microscopic field are presented. B, Quantitative analysis of the cell infiltration area in myocardium. There is no significant difference between the untreated group and the treated group. C, Quantitative analysis of the fibrotic area in myocardium. The untreated group showed severe fibrosis, however, the treated group showed a smaller fibrotic area. *P < 0.05, Fisher’s PLSD. Values are mean ± SEM. Scale bars = 1mm

The fibrosis area ratio of myocardium in the treated group (2.0 ± 0.1%) was lower than the untreated group (4.6 ± 1.0%, P<0.05). Regarding the cell infiltration area in myocardium, there was no significant difference between the treated group (1.75 ± 0.57) and the untreated group (3.04 ± 0.58) (p = 0.27). (Fig. 1)

### Immunohistochemistry

Immunohistochemical analysis indicated that there was no significant difference in the number of CD4 positive cells per entire area (mm^2^) between the treated group (9.86 ± 5.26) and the untreated group (7.93 ± 2.31). Similarly, there was no significant difference in the number of F4/80 positive cells per entire area (mm^2^) between the treated group (21.8 ± 8.8) and the untreated group (20.9 ± 6.7).

### RT-PCR

**Fig 2:**
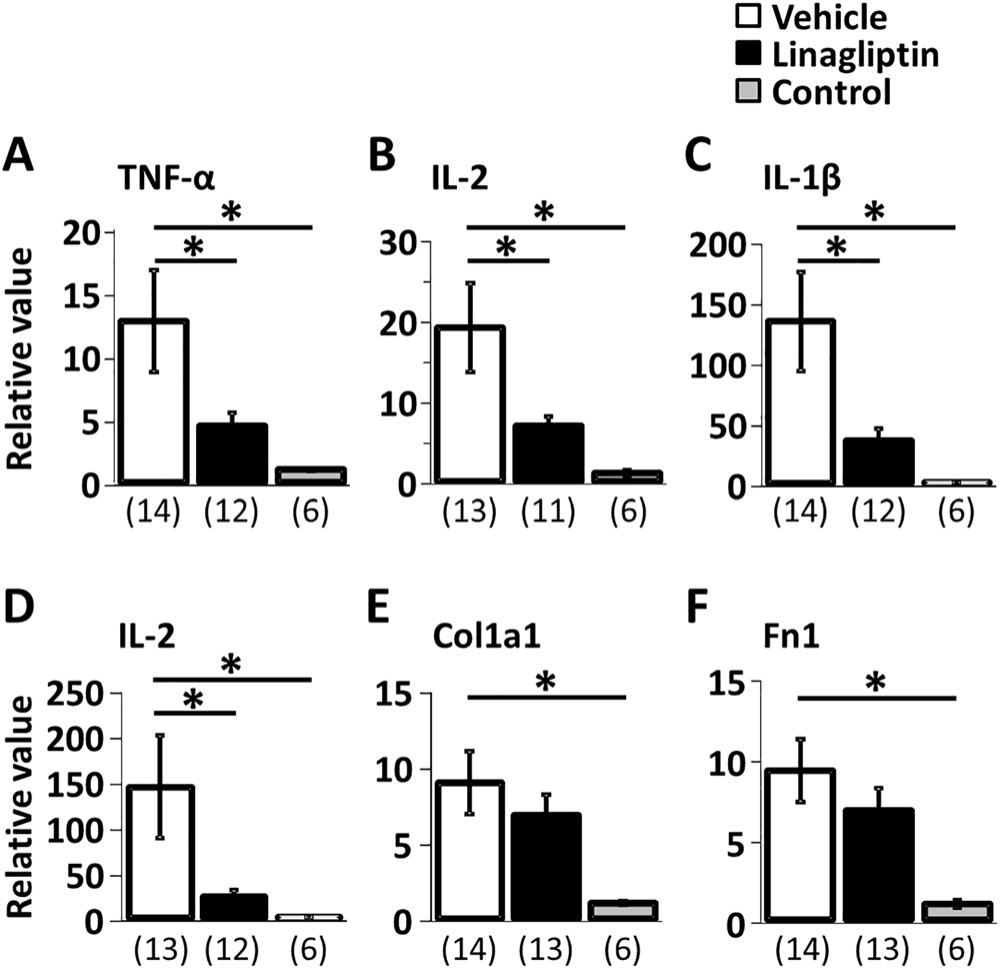
Results of Quantitative PCR analysis. mRNA expression of TNF-α (A), IL-2 (B), IL-1β (C), IL-6 (D), Col-1a1 (E) and Fn-1(F) in the hearts on day 21. *P < 0.05, Fisher’s PLSD. Values are mean ± SEM.

Quantitative PCR analysis showed that the levels of mRNA expression of IL-2, TNF-α, IL-1β and IL-6 were significantly lower in the linagliptin treated group than in the untreated EAM group on day 21. Furthermore, linagliptin tended to reduce the mRNA levels of Col-1a1 and Fn-1 compared to the untreated EAM group. (Fig. 2)

### Lymphocyte Proliferation Assay

**Fig 3:**
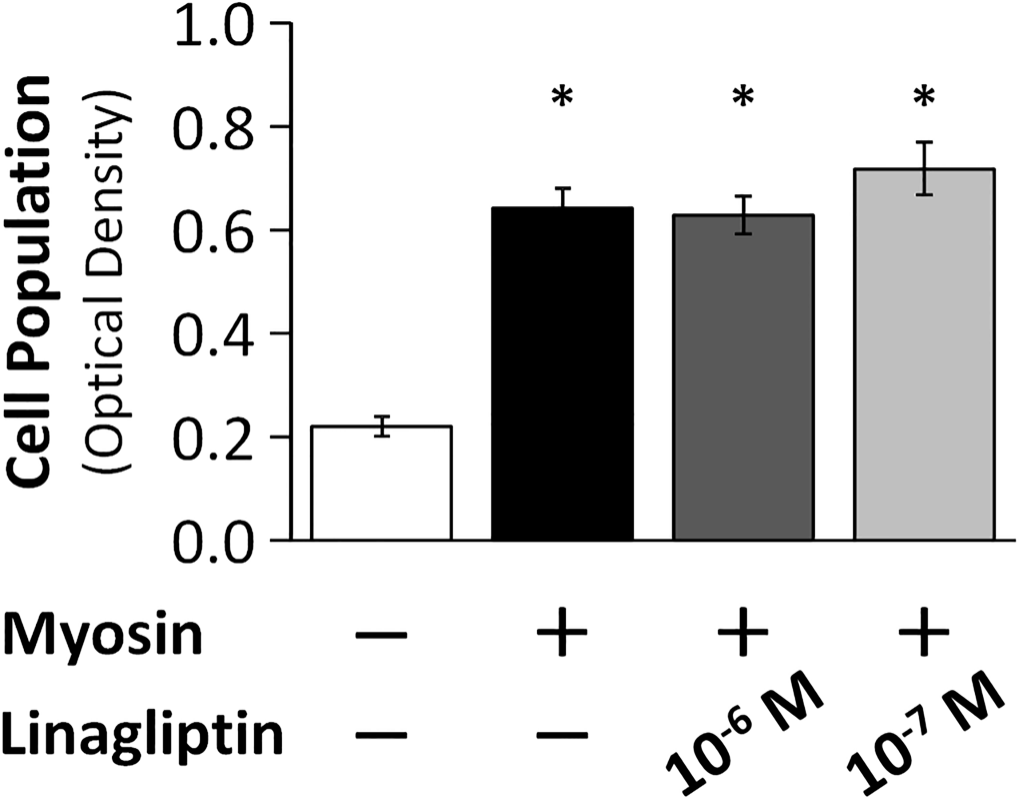
Results of lymphocyte proliferation assay. Cultures with spleen-derived lymphocytes obtained from EAM mice were incubated at 37°C with 5% CO_2_ for 70 hours. Lymphocyte proliferation was evaluated. Treatment of a DPP-4 inhibitor did not affect antigen-induced spleen-derived lymphocyte proliferation. *P < 0.05 vs. Myosin-, Linagliptin-, Bonferroni comparison test. Values are mean ± SEM.

We performed a lymphocyte proliferation assay to examine the effect of a DPP-4 inhibitor on antigen-induced spleen cell proliferation (n = 12 per group). The DPP-4 inhibitor did not affect antigen-induced lymphocyte proliferation. (Fig. 3)

## Discussion

Although DPP-4 inhibitors are known to have favorable effects on cardiovascular disease, such as aortic aneurysm and stroke [8, 9], the effect of DPP-4 inhibitors on myocarditis has not been investigated. Our study is the first to show the anti-fibrotic effects of a DPP-4 inhibitor (linagliptin) on EAM mice.

A large amount of evidence suggests that T helper 1 (Th1) cytokines (e.g., IL-2 and TNF-α) are involved in the progression of EAM [16, 17]. In particular, TNF-α has an important role in the pathogenesis of EAM [4, 18, 19]. Blockade of TNF-α ameliorated inflammation in EAM mice [4]. Liu et al. demonstrated that TNF-α plays a critical role in the degradation of myocardial extracellular matrix components through upregulating matrix metalloproteinases, which in turn deteriorates myocardial contractility [18]. Myocardial fibrosis was markedly increased in transgenic mice with cardiac-specific overexpression of TNF-α [20]. We also demonstrated that administration of linagliptin effectively suppressed fibrosis and reduced IL-1β and IL-6 in EAM mice. Because these cytokines are known to be critical factors in the acceleration of EAM [11, 21], linagliptin has an ability to suppress EAM development. Furthermore, Col-1a1 and Fn-1, which are known to be pro-fibrotic factors in injured tissues, were suppressed by linagliptin. These data suggest that linagliptin may have a pleiotropic effect on anti-inflammation and anti-fibrosis. Recent papers revealed that DPP-4 inhibitors have an inhibitory effect on fibroblast activity. Kanasaki et al. showed that linagliptin ameliorated kidney fibrosis in diabetic mice without altering blood glucose levels. The therapeutic effects of linagliptin on diabetic kidneys were associated with the suppression of profibrotic programs [22]. At this moment we cannot reveal the detailed mechanisms, however, linagliptin may have a novel pleiotropic action that suppresses inflammation and tissue fibrosis.

The soluble form of DPP-4, which lacks transmembrane domain functions as an aminopeptidase, is described above. On the other hand, DPP-4, also known as CD26, is expressed on the surface of immune cells, including T cells, B cells, NK cells, dendritic cells and macrophages [6, 23, 24]. DPP-4 interacts with a range of ligands, such as adenosine deaminase and kidney Na+/H+ ion exchanger [6], thereby playing an important role in cellular migration and cellular adhesion to collagen type I and to fibronectin. Shin et al. demonstrated that cell migration and adhesion via DPP-4 were independent of enzymatic activity of DPP-4 [25]. Furthermore, CD26 is also involved in the costimulatory interaction in T cell response to antigens [26–28]. However, our current results showed the antigen-induced lymphocyte proliferation was not suppressed by a DPP-4 inhibitor. It suggests that DPP-4 mediated signaling may not be involved in the proliferation of lymphocytes during inflammatory responses.

To understand the molecular mechanism of DPP-4 in the progression of inflammation-mediated cardiovascular diseases, the substrates of DPP-4 must be taken into account. DPP-4 is known to have various substrates which are inactivated by the activity of aminopeptidase, including GLP-1, GIP and SDF-1α [6, 29]. Bose et al. demonstrated that GLP-1 has a cardioprotective function in a rat model of myocardial ischemia and reperfusion injury [30]. Exenatide, a GLP-1 analogue, reduced myocardial infarct size in a pig model of myocardial ischemia [7]. Shigeta et al. revealed that inhibition of DPP-4 reversed diastolic LV dysfunction in response to pressure overload through DPP-4/SDF-1α-mediated local actions upon angiogenesis [29]. Thus, DPP-4 inhibitors may have some therapeutic effects on EAM through modulating the function of DPP-4 substrates.

## Limitation

Our current study revealed that the DPP-4 inhibitor significantly decreased the levels of IL-2, TNF-α, IL-1β and IL-6 mRNA expression and the fibrosis in the EAM hearts. However, our study did not clarify whether the DPP-4 inhibitor suppresses the function of lymphocytes directly and whether the substrate of DPP-4 is related to the anti-inflammatory effect in EAM. As various factors, such as virus infection and autoimmunity, are related to myocarditis [4, 31], the effects of DPP-4 inhibitors should also be elucidated through the use of a viral myocarditis model. Thus, further research is needed to clarify the effect of DPP-4 inhibitors on murine EAM.

## Conclusions

A DPP-4 inhibitor, linagliptin, prevented cardiac fibrosis and attenuated Th1 cytokine expression in EAM. This finding suggests the possibility of using a DPP-4 inhibitor to alleviate fibrosis in patients with myocarditis.

## Supporting Information

S1 Dataset (XLSX)

S2 Dataset (XLSX)

S3 Dataset (XLS)

S4 Dataset (XLS)

S5 Dataset (XLS)

S6 Dataset (XLS)

S7 Dataset (XLS)

S8 Dataset (XLS)

S9 Dataset (XLS)

## References

[1] Sagar S, Liu PP, Cooper LT Jr. (2012) Myocarditis. Lancet 379: 738–747. 10.1016/S0140-6736(11)60648-X 22185868 PMC5814111

[2] Suzuki J, Ogawa M, Maejima Y, Isobe K, Tanaka H, Sagesaka YM, et al (2007) Tea catechins attenuate chronic ventricular remodeling after myocardial ischemia in rats. J Mol Cell Cardiol 42: 432–440. 17174976 10.1016/j.yjmcc.2006.10.006

[3] Kishimoto C, Nimata M, Okabe TA, Shioji K (2013) Immunoglobulin treatment ameliorates myocardial injury in experimental autoimmune myocarditis associated with suppression of reactive oxygen species. Int J Cardiol 167: 140–145. 10.1016/j.ijcard.2011.12.058 22244481

[4] Leuschner F, Katus HA, Kaya Z (2009) Autoimmune myocarditis: past, present and future. J Autoimmun 33: 282–289. 10.1016/j.jaut.2009.07.009 19679447

[5] Matteucci E, Giampietro O (2009) Dipeptidyl peptidase-4 (CD26): knowing the function before inhibiting the enzyme. Curr Med Chem 16: 2943–2951. 19689275 10.2174/092986709788803114

[6] Zhong J, Rao X, Rajagopalan S (2013) An emerging role of dipeptidyl peptidase 4 (DPP4) beyond glucose control: potential implications in cardiovascular disease. Atherosclerosis 226: 305–314. 10.1016/j.atherosclerosis.2012.09.012 23083681

[7] Timmers L, Henriques JP, de Kleijn DP, Devries JH, Kemperman H, Steendijk P, et al (2009) Exenatide reduces infarct size and improves cardiac function in a porcine model of ischemia and reperfusion injury. J Am Coll Cardiol 53: 501–510. 10.1016/j.jacc.2008.10.033 19195607

[8] Bao W, Morimoto K, Hasegawa T, Sasaki N, Yamashita T, Hirata K, et al (2013) Orally administered dipeptidyl peptidase-4 inhibitor (alogliptin) prevents abdominal aortic aneurysm formation through an antioxidant effect in rats. J Vasc Surg. 10.1016/j.jvs.2013.04.048 23790558

[9] Darsalia V, Ortsater H, Olverling A, Darlof E, Wolbert P, Nystrom T, et al (2013) The DPP-4 inhibitor linagliptin counteracts stroke in the normal and diabetic mouse brain: a comparison with glimepiride. Diabetes 62: 1289–1296. 10.2337/db12-0988 23209191 PMC3609599

[10] Pummerer CL, Luze K, Grassl G, Bachmaier K, Offner F, Burrell SK, et al (1996) Identification of cardiac myosin peptides capable of inducing autoimmune myocarditis in BALB/c mice. J Clin Invest 97: 2057–2062. 8621795 10.1172/JCI118642 PMC507280

[11] Eriksson U, Kurrer MO, Schmitz N, Marsch SC, Fontana A, Eugster HP, et al (2003) Interleukin-6-deficient mice resist development of autoimmune myocarditis associated with impaired upregulation of complement C3. Circulation 107: 320–325. 12538435 10.1161/01.cir.0000043802.38699.66

[12] Thomas L, Eckhardt M, Langkopf E, Tadayyon M, Himmelsbach F, Mark M (2008) (R)-8-(3-amino-piperidin-1-yl)-7-but-2-ynyl-3-methyl-1-(4-methyl-quinazolin-2-ylm ethyl)-3,7-dihydro-purine-2,6-dione (BI 1356), a novel xanthine-based dipeptidyl peptidase 4 inhibitor, has a superior potency and longer duration of action compared with other dipeptidyl peptidase-4 inhibitors. J Pharmacol Exp Ther 325: 175–182. 10.1124/jpet.107.135723 18223196

[13] Futamatsu H, Suzuki J, Kosuge H, Yokoseki O, Kamada M, Ito H, et al (2003) Attenuation of experimental autoimmune myocarditis by blocking activated T cells through inducible costimulatory molecule pathway. Cardiovasc Res 59: 95–104. 12829180 10.1016/s0008-6363(03)00334-1

[14] Suzuki J, Ogawa M, Futamatsu H, Kosuge H, Tanaka H, Isobe M (2006) A cyclooxygenase-2 inhibitor alters Th1/Th2 cytokine balance and suppresses autoimmune myocarditis in rats. J Mol Cell Cardiol 40: 688–695. 16499924 10.1016/j.yjmcc.2006.01.006

[15] Futamatsu H, Suzuki J, Mizuno S, Koga N, Adachi S, Kosuge H, et al (2005) Hepatocyte growth factor ameliorates the progression of experimental autoimmune myocarditis: a potential role for induction of T helper 2 cytokines. Circ Res 96: 823–830. 15774858 10.1161/01.RES.0000163016.52653.2e

[16] Daniels MD, Hyland KV, Wang K, Engman DM (2008) Recombinant cardiac myosin fragment induces experimental autoimmune myocarditis via activation of Th1 and Th17 immunity. Autoimmunity 41: 490–499. 10.1080/08916930802167902 18781477 PMC2702149

[17] Watanabe R, Azuma RW, Suzuki JI, Ogawa M, Itai A, Hirata Y, et al (2013) Inhibition of NF-kappaB activation by a novel IKK inhibitor reduces the severity of experimental autoimmune myocarditis via suppression of T-cell activation. Am J Physiol Heart Circ Physiol. 10.1152/ajpheart.00159.2013 24097428

[18] Liu X, Li B, Wang W, Zhang C, Zhang M, Zhang Y, et al (2012) Effects of HMG-CoA reductase inhibitor on experimental autoimmune myocarditis. Cardiovasc Drugs Ther 26: 121–130. 10.1007/s10557-012-6372-6 22382902

[19] Tang Z, McGowan BS, Huber SA, McTiernan CF, Addya S, Surrey S, et al (2004) Gene expression profiling during the transition to failure in TNF-alpha over-expressing mice demonstrates the development of autoimmune myocarditis. J Mol Cell Cardiol 36: 515–530. 15081311 10.1016/j.yjmcc.2004.01.008

[20] Sivasubramanian N, Coker ML, Kurrelmeyer KM, MacLellan WR, DeMayo FJ, Spinale FG, et al (2001) Left ventricular remodeling in transgenic mice with cardiac restricted overexpression of tumor necrosis factor. Circulation 104: 826–831. 11502710 10.1161/hc3401.093154

[21] Zempo H, Sugita Y, Ogawa M, Watanabe R, Suzuki JI, Isobe M (2014) A P2X7 receptor antagonist attenuates experimental autoimmune myocarditis via suppressed myocardial CD4 T and macrophage infiltration and NADPH oxidase 2/4 expression in mice. Heart Vessels. 10.1007/s00380-014-0527-2 24879505

[22] Kanasaki K, Shi S, Kanasaki M, He J, Nagai T, Nakamura Y, et al (2014) Linagliptin-mediated DPP-4 inhibition ameliorates kidney fibrosis in streptozotocin-induced diabetic mice by inhibiting endothelial-to-mesenchymal transition in a therapeutic regimen. Diabetes 63: 2120–2131. 10.2337/db13-1029 24574044

[23] Reinhold D, Bank U, Buhling F, Kahne T, Kunt D, Faust J, et al (1994) Inhibitors of dipeptidyl peptidase IV (DP IV, CD26) specifically suppress proliferation and modulate cytokine production of strongly CD26 expressing U937 cells. Immunobiology 192: 121–136. 7750986 10.1016/S0171-2985(11)80412-2

[24] Zhong J, Rao X, Deiuliis J, Braunstein Z, Narula V, Hazey J, et al (2013) A potential role for dendritic cell/macrophage-expressing DPP4 in obesity-induced visceral inflammation. Diabetes 62: 149–157. 10.2337/db12-0230 22936179 PMC3526020

[25] Shin JW, Jurisic G and Detmar M (2008) Lymphatic-specific expression of dipeptidyl peptidase IV and its dual role in lymphatic endothelial function. Exp Cell Res 314: 3048–3056. 10.1016/j.yexcr.2008.07.024 18708048 PMC3398155

[26] Ohnuma K, Uchiyama M, Hatano R, Takasawa W, Endo Y, Dang NH, et al (2009) Blockade of CD26-mediated T cell costimulation with soluble caveolin-1-Ig fusion protein induces anergy in CD4+T cells. Biochem Biophys Res Commun 386: 327–332. 10.1016/j.bbrc.2009.06.027 19523449

[27] Martin M, Huguet J, Centelles JJ, Franco R (1995) Expression of ecto-adenosine deaminase and CD26 in human T cells triggered by the TCR-CD3 complex. Possible role of adenosine deaminase as costimulatory molecule. J Immunol 155: 4630–4643. 7594462

[28] Pacheco R, Martinez-Navio JM, Lejeune M, Climent N, Oliva H, Gatell JM, et al (2005) CD26, adenosine deaminase, and adenosine receptors mediate costimulatory signals in the immunological synapse. Proc Natl Acad Sci U S A 102: 9583–9588. 15983379 10.1073/pnas.0501050102 PMC1172240

[29] Shigeta T, Aoyama M, Bando YK, Monji A, Mitsui T, Takatsu M, et al (2012) Dipeptidyl peptidase-4 modulates left ventricular dysfunction in chronic heart failure via angiogenesis-dependent and-independent actions. Circulation 126: 1838–1851. 10.1161/CIRCULATIONAHA.112.096479 23035207

[30] Bose AK, Mocanu MM, Carr RD, Brand CL, Yellon DM (2005) Glucagon-like peptide 1 can directly protect the heart against ischemia/reperfusion injury. Diabetes 54: 146–151. 15616022 10.2337/diabetes.54.1.146

[31] Shauer A, Gotsman I, Keren A, Zwas DR, Hellman Y, Durst R, et al (2013) Acute viral myocarditis: current concepts in diagnosis and treatment. Isr Med Assoc J 15: 180–185. 23662385

